# Daclatasvir combined with peginterferon-α and ribavirin for the treatment of chronic hepatitis C: a meta-analysis

**DOI:** 10.1186/s40064-016-3218-x

**Published:** 2016-09-15

**Authors:** Qin Peng, Kang Li, Ming Rong Cao, Cai Qun Bie, Hui Jun Tang, Shao Hui Tang

**Affiliations:** 1 Department of Gastroenterology, The First Affiliated Hospital, Jinan University, 613 Huang Pu Avenue, Guangzhou, China; 2 Department of Gastroenterology, The Affiliated HeXian Memorial Hospital, Southern Medical University, Guangzhou, China; 3 Department of General Surgery, The First Affiliated Hospital, Jinan University, Guangzhou, Guangdong China; 4 Department of Gastroenterology, The Affiliated Shenzhen Shajing Hospital, Guangzhou Medical University, Shenzhen, 518104 China

**Keywords:** Chronic, Daclatasvir, Hepatitis C, Meta-analysis, Randomized controlled trials

## Abstract

Daclatasvir, a HCV NS5A inhibitor, is a new direct-acting antiviral drug for chronic hepatitis C (CHC). This study aimed to evaluate the efficacy and safety of daclatasvir combined with peginterferon-α (pegIFN-α) and ribavirin (RBV) for the treatment of CHC. The databases of PUBMED, EMBASE, COCHRANE, WANFANG, and CNKI were retrieved to identify eligible studies. Pooled risk ratio (RR) and 95 % confidence interval (CI) were calculated using random or fixed models. A total of six RCTs including 1100 adult patients with CHC met the inclusion criteria and the patients were infected with HCV genotype 1–4, with the genotype 1 infection accounting for 73.1 %. Meta-analysis showed daclatasvir-based combination therapy yielded a significantly higher probability of achieving the overall RVR (46.43 vs. 18.97 %) with pooled RR of 3.77 (95 % CI 1.95–7.28, *p* < 0.0001) and a slightly higher probability of achieving the overall SVR_24_ (65.08 vs. 47.77 %) with pooled RR of 1.41 (95 % CI 1.18–1.68, *p* < 0.0001), and did not show increased adverse events compared with the pegIFN-α/RBV regimen (control group). Subgroup analysis showed the rate of RVR and SVR_24_ in high-dose daclatasvir (60 mg/day) group were slightly higher than the overall results; the rate of RVR in low-dose daclatasvir (10 mg/day) group was also higher than the control group, but its SVR_24_ rate was similar between the two groups. Daclatasvir combined with pegIFN-α/RBV is effective and safe in treating adult patients with CHC, especially HCV genotype 1 infection, and daclatasvir (60 mg/day) is a better choice as compared with daclatasvir (10 mg/day).

## Background

Chronic hepatitis C virus (HCV) infection is common worldwide, affecting approximately 185 million people globally, and is the cause of 704,000 deaths annually (World Health Organization 2016; Gower et al. 2014; Chen and Ma 2014). About 75–85 % of all patients contracting HCV will progress to chronic infection, with 15–25 % of patients spontaneously clearing the infection (Centers for Disease Control and Prevention 2014). The progression between various stages of fibrosis can take decades, and often patients are asymptomatic until end-stage disease. Approximately one-third of patients with chronic HCV infection will develop cirrhosis or hepatocellular carcinoma (World Health Organization 2016). So more and more attentions are paid to the early detection and treatment of chronic hepatitis C (CHC). The goal of treatment of CHC is to reduce associated morbidity and mortality and to improve health-related quality of life (AASLD/IDSA/IAS–USA 2014; American Association for the Study of the Liver 2016). Achievement of sustained virological response (SVR) is a surrogate endpoint for these goals (Ghany et al. 2009a, b; European Association for the Study of the Liver 2014a, b). Pegylated interferon-α (pegIFN-α) in combination with ribavirin (RBV) has always been the current standard of care for CHC, which result in remarkable biochemical and histological improvements in the liver (Chen and Li 2015; European Association for the Study of the Liver 2014a, b). However, patients infected with HCV genotype 1 have experienced a poor response to this therapy as observed by SVR rates of only 40–50 % (Chinese Society of Hepatology et al. 2015).

In recent years, the direct antiviral agents (DAA) have gradually emerged, which include the nonstructural protein 3/4A (NS3/4A) protease inhibitor, the NS5A inhibitor, the NS5B polymerase inhibitor, the NS4B protease inhibitor, and the NS3 protease inhibitor, etc (Wan et al. 2014). The emergence of these drugs has brought new choice for the treatment. Daclatasvir is a first-in-class, potent, and highly selective NS5A replication complex inhibitor with broad genotypic coverage (genotypes 1–5) and a pharmacokinetic profile supportive of once-daily dosing (Degasperi et al. 2015; Manolakopoulos et al. 2016; Keating 2016). Nettles et al. (2011) conducted the first randomized controlled trial (RCT), showing that daclatasvir in combination with pegIFN-α/RBV achieved satisfactory therapeutic effects. Subsequently, other studies with different design, location, and population further examined the efficacy of daclatasvir in the treatment of CHC. Here, we combined all published RCTs on this issue to quantitatively assess the efficacy and safety of daclatasvir combined with pegIFN-α and ribavirin in the treatment of CHC.

## Methods

### Literature search

A systematic search of PUBMED, EMBASE, COCHRANE, WANFANG, and CNKI (Chinese database) was performed with no year restrictions. The search strategy included the following terms: ‘HCV’ (e.g. ‘hepatitis C’, ‘hepatitis C virus’, ‘HCV infection’); ‘daclatasvir’ (e.g. ‘BMS-790052’); ‘RCT’ (e.g. ‘randomized controlled trial’). We carried out a second retrieval for the references of review.

### Inclusion and exclusion criteria

Trials were considered for inclusion if they met the following four criteria. (a) The research type was RCT design. (b) This study was conducted in patients aged 18–70 years who had chronic HCV infection over 6 months with positive anti-HCV. Additional inclusion criteria included HCV-RNA ≥ 10^5^ IU/ml. Meanwhile, serum urea nitrogen, creatinine and prothrombin activity were at normal levels. (c) Patients in experimental group were treated with daclatasvir in combination with peg-IFN/RBV; while patients in control group were treated with placebo in combination with peg-IFN/RBV. (d) The Jadad score of RCTs should not be less than three points.

**Fig. 1:**
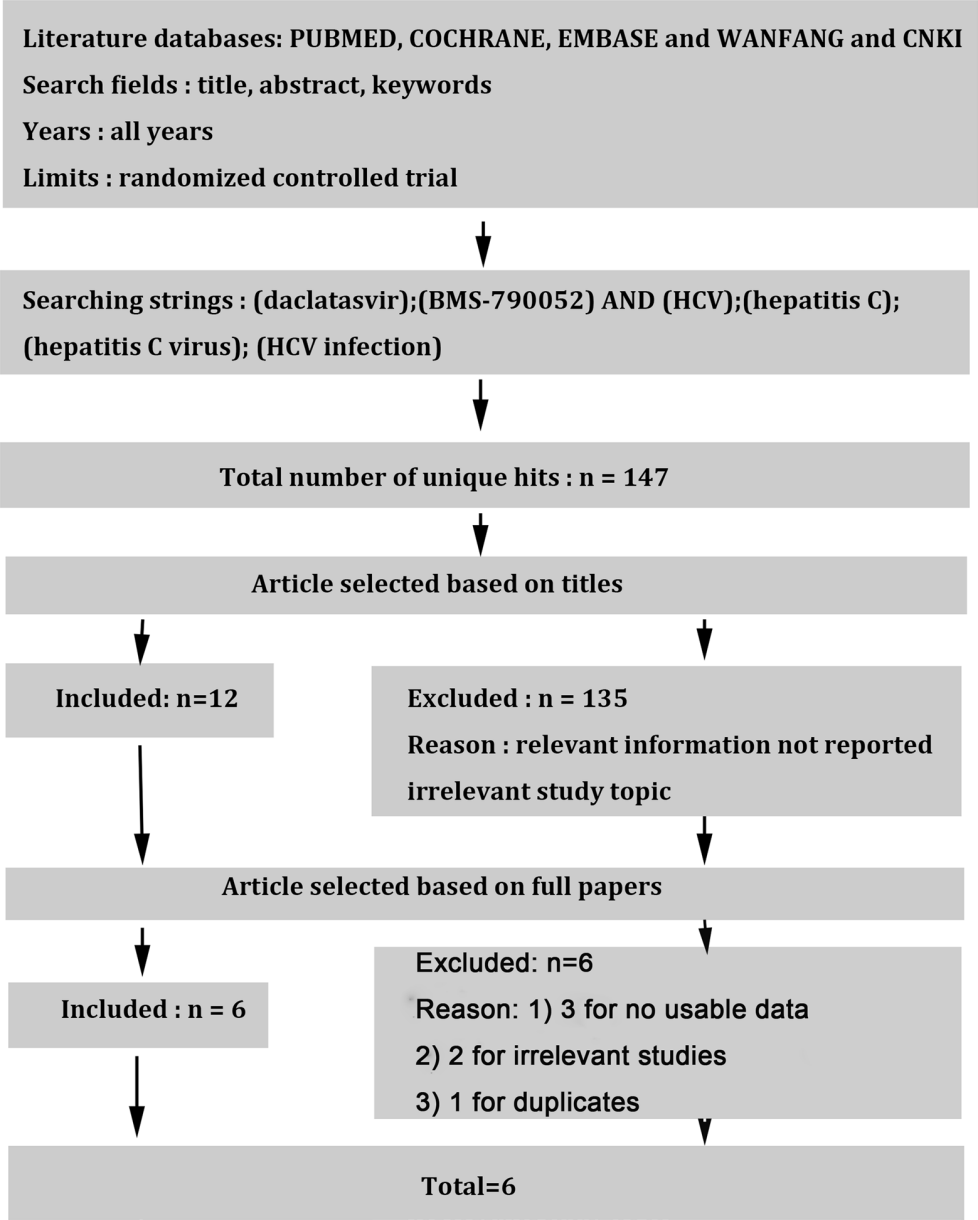
Search strategy and flow of information relative to the meta-analysis

Trials were excluded if: (a) the study belonged to non-randomized controlled trial (NRCT) or the Jadad score of RCTs was less than three points; (b) the study didn’t provide the outcome or the measurement; (c) the study was literature review or repeated other reports (Fig. 1).

### Data extraction

Data extracted from each study included published information (including the first author and published time); subjects (including the number of patients, gender, age, HCV-RNA content and viral genotype); treatment protocols (including the drugs used in experimental group and control group, dosage regimen, course of the treatment, efficient individual quantity).

### Quality assessment

We conducted a risk of bias assessment based on Jadad scores (Jadad et al. 1996): (a) method of randomization was mentioned; (b) method of randomization was appropriate; (c) the trial was double-blind; (d) method of blinding was identical placebo; (e) there was a description of withdraws and drop outs. If the study complied with any of the above description, it would get one point. The study with total score above 3 was divided into high-quality research.

### Statistical analysis

Statistical heterogeneity between studies was examined using the *I*^2^ value. *I*^2^ ranges of 25–<50, 50–<75 and ≥75 % were considered to represent low, moderate, and high heterogeneity, respectively. If heterogeneity is low, we choose fixed-effects model, and if heterogeneity is moderate or high, we choose random-effects model (Tian et al. 2013). Sensitive analysis was performed to examine the reliability of the results by omitting one study each time and recalculating the pooled RR. Publication bias was evaluated by visual inspection of Begg’s funnel plot and tested by the Begg’s test (significant at *p* < 0.1). In addition, the trim-and-fill method was used to adjust the pooled RR and 95 % CI if observed publication bias existed. All statistical analyses were completed using Stata statistical software version 12.0.

## Results

### Study characteristics

A total of 147 potentially relevant studies were identified through the electronic search. Through a review of titles and abstracts, 12 articles were chosen for full review. Six studies retrieved for further review were excluded. The remaining six studies met all criteria and were included in this meta-analysis. Baseline characteristics of included studies were presented in Table 1. Among these papers, there were 926 cases treated with daclatasvir in combination with peg-IFN-α/RBV (daclatasvir group) and 174 cases treated with peg-IFN-α/RBV (control group) and they were infected with HCV genotype 1–4 infection with the genotype 1 infection accounting for 73.1 %, published from 2012 to 2015. The sample sizes of these studies ranged from 42 to 419. Median age of the patients ranged 50–57 years. The percentage of men in each treatment arm ranged 22.0–75.0 %. The quality assessment of all RCTs were greater than or equal to 4, which indicated that all included articles were high quality (Table 2).

**Table 1:** Characteristics of the included studies

Study	Median age (range)	Gender (male, %)	HCV-RNA (median log_10_)	Genotype	Type of treatment and dose	Treatment duration (week)	RVR	SVR_24_	Relapse	VB	TDAE	SAE
Hézode et al. (2015) (n = 395)	51 (22–70)	67.3	6.5	G1a, 275/395	D (20 mg/day) + P (180 μg/week) + R (1.0–1.2 g/day)	24	91 (57)	95 (60)	24 (15)	14 (9)	7 (4)	12 (8)
	50 (18–67)	65.2	6.5	G1b, 88/395	D (60 mg/day) + P (180 μg/week) + R (1.0–1.2 g/day)	24	87 (55)	99 (63)	22 (14)	15 (9)	7 (4)	13 (8)
	51 (25–66)	70.5	6.4	G4, 32/395	PBO + P (180 μg/week) + R (1.0–1.2 g/day)	24	11 (14)	30 (38)	9 (12)	2 (3)	8 (10)	6 (8)
Dore et al. (2015) (n = 151)	52 (28–64)	54.2	6.4	G2, 71/151	D (60 mg/day) + P (180 μg/week) + R (0.8 g/day)	12	43 (86)	38 (76)	0 (0)	8 (16)	4 (8)	4 (8)
	52 (25–67)	65.2	6.6	G3, 80/151	D (60 mg/day) + P (180 μg/week) + R (0.8 g/day)	16	35 (70)	37 (74)	0 (0)	7 (14)	3 (6)	0 (0)
	55 (20–63)	45.8	6.6		PBO + P (180 μg/week) + R (0.8 g/day)	24	20 (39)	31 (61)	2 (4)	5 (10)	2 (4)	3 (6)
Suzuki et al. (2014) (n = 45)	51 (21–68)	22.0	6.7	G1, 45/45	D (10 mg/day) + P (60–150 μg/week) + R (0.6–1 g/day)	24/48	12 (67)	8 (44)	4 (22)	5 (28)	1 (6)	1 (6)
	55 (36–70)	60.0	6.7		D (60 mg/day) + P (60–150 μg/week) + R (0.6–1 g/day)	24/48	11 (58)	12 (63)	3 (16)	4 (21)	1 (5)	0 (0)
	50 (42–66)	50.0	6.9		PBO + P (60–150 μg/week) + R (0.6–1 g/day)	48	0 (0)	5 (63)	2 (25)	1 (13)	0 (0)	0 (0)
Izumi et al. (2014) (n = 42)	56 (26–68)	44.0	6.8	G1, 42/42	D (10 mg/day) + P (180 μg/week) + R (0.6–1 g/day)	24	12 (71)	12 (71)	3 (18)	2 (12)	1 (6)	2 (12)
	57 (31–67)	25.0	6.6		D (60 mg/day) + P (180 μg/week) + R (0.6–1 g/day)	24	13 (76)	15 (88)	1 (6)	1 (6)	2 (12)	0 (0)
	54 (41–65)	38.0	6.5		PBO + P (180 μg/week) + R (0.6–1 g/day)	24	1 (13)	6 (75)	1 (13)	0 (0)	0 (0)	0 (0)
Pol et al. (2012) (n = 48)	52 (38–66)	75.0	6.3	G1a, 32/48	D (3 mg/day) + P (180 μg/week) + R (1.0–1.2 g/day)	12	5 (42)	5 (42)	2 (17)	2 (17)	1 (8)	1 (8)
	51 (37–68)	67.0	6.4		D (10 mg/day) + P (180 μg/week) + R (1.0–1.2 g/day)	12	11 (92)	10 (83)	1 (8)	0 (0)	1 (8)	1 (8)
	51 (43–67)	58.0	6.5	G1b, 16/48	D (60 mg/day) + P (180 μg/week) + R (1.0–1.2 g/day)	12	10 (83)	10 (83)	1 (8)	1 (8)	4 (33)	1 (8)
	50 (28–67)	67.0	6.7		PBO + P (180 μg/week) + R (1.0–1.2 g/day)	12	1 (8)	3 (25)	5 (42)	0 (0)	2 (17)	0 (0)
Ratziu et al. (2012) (n = 419)	NR	NR	NR	NR	D (20 mg/day) + P/R	12	47 (23)	NR	NR	NR	NR	12 (6)
					D (60 mg/day) + P/R	12	53 (27)					10 (5)
					PBO + P/R	12	0 (0)					3 (18)

The data of RVR, SVR24, relapse, VB, TDAE and SAE are presented as n (%)

Median age, gender, HCV-RNA, treatment duration, RVR, cEVR and SVR24 is respectively divided into different groups according to the type of treatment and dose; Genotype is divided into different groups according to G1a, G1b, G2, G3 and G4

*D* daclatasvir, *P* pegylated interferon-α, *R* ribavirin, *PBO* placebo, *RVR* rapid virological response, *SVR*_*24*_ sustained virological response at post-treatment week 24, *VB* virological breakthrough, *TDAE* treatment discontinuation due to an adverse event, *SAE* serious adverse event, *NR* not reported

**Table 2:** Jadad score of clinical trials

Trial	Randomization	Double-blinding	Withdraw and drop out	Jadad score
Hézode et al. (2015)	Method of randomization was mentioned and it was appropriate (2)	Method of blinding was identical placebo (2)	There was a description of withdraws and drop outs (1)	5
Dore et al. (2015)	Method of randomization was mentioned and it was appropriate (2)	Method of blinding was identical placebo (2)	There was a description of withdraws and drop outs (1)	5
Suzuki et al. (2014)	Method of randomization was mentioned and it was appropriate (2)	Method of blinding was identical placebo (2)	There was a description of withdraws and drop outs (1)	5
Izumi et al. (2014)	Method of randomization was mentioned and it was appropriate (2)	Method of blinding was identical placebo (2)	There was a description of withdraws and drop outs (1)	5
Pol et al. (2012)	Method of randomization was mentioned but not described (1)	Method of blinding was identical placebo (2)	There was a description of withdraws and drop outs (1)	4
Ratziu et al. (2012)	Method of randomization was mentioned but not described (1)	Method of blinding was identical placebo (2)	There was a description of withdraws and drop outs (1)	4

### Overall analysis

#### Rapid virological response (RVR)

**Fig. 2:**
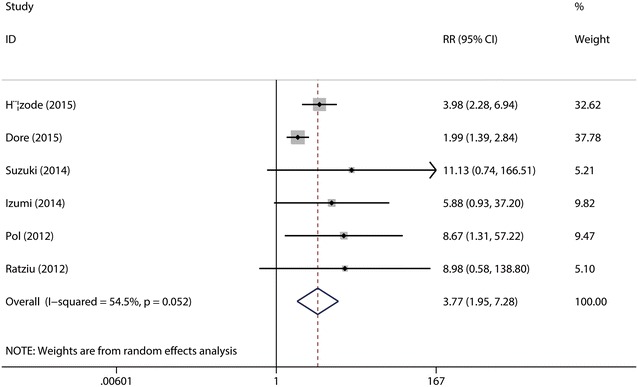
Forest plot of RVR rate of DCV + P/R and PBO + P/R for CHC

RVR was defined as an undetectable HCV-RNA level at 4 weeks after treatment initiation. A total of the six studies (Hézode et al. 2015; Dore et al. 2015; Suzuki et al. 2014; Izumi et al. 2014; Pol et al. 2012; Ratziu et al. 2012) were included in this combined analysis. The test for the heterogeneity among the studies showed statistical significance (*I*^2^ = 54.5 %), so the random-effects model was used. Meta-analysis results suggested that the overall RVR rate was significantly higher in daclatasvir group (46.43 %) as compared with that in control group (18.97 %) (RR = 3.77, 95 % CI 1.95–7.28, *p* < 0.0001, Fig. 2). Sensitive analysis showed that no individual studies could change the pooled results. Publication bias did not exist (*p* = 0.851) when the Begger test was performed.

#### Sustained virological response at post-treatment week 24 (SVR_24_)

**Fig. 3:**
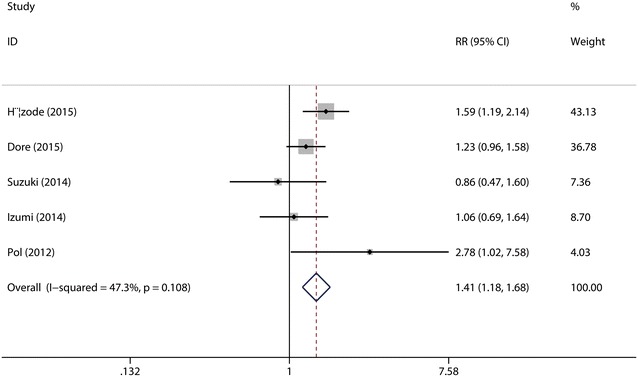
Forest plot of SVR rate of DCV + P/R and PBO + P/R for CHC

SVR_24_, defined as an undetectable viral load at the end of treatment and 24 weeks after the end of treatment, historically has been regarded as a virologic cure. A total of the five studies (Hézode et al. 2015; Dore et al. 2015; Suzuki et al. 2014; Izumi et al. 2014; Pol et al. 2012) were included in this combined analysis. The test for the heterogeneity showed that there was no statistical significance (*I*^2^ = 47.3 %), so the fixed-effects model was used. Meta-analysis results revealed that the overall SVR_24_ rate was significantly higher in daclatasvir group (65.08 %) as compared with that in control group (47.77 %) (RR = 1.41, 95 % CI 1.18–1.68, *p* < 0.0001, Fig. 3). Sensitive analysis showed that no individual studies could change the pooled results. Publication bias did not exist (*p* = 0.624) when the Begger test was performed.

**Fig. 4:**
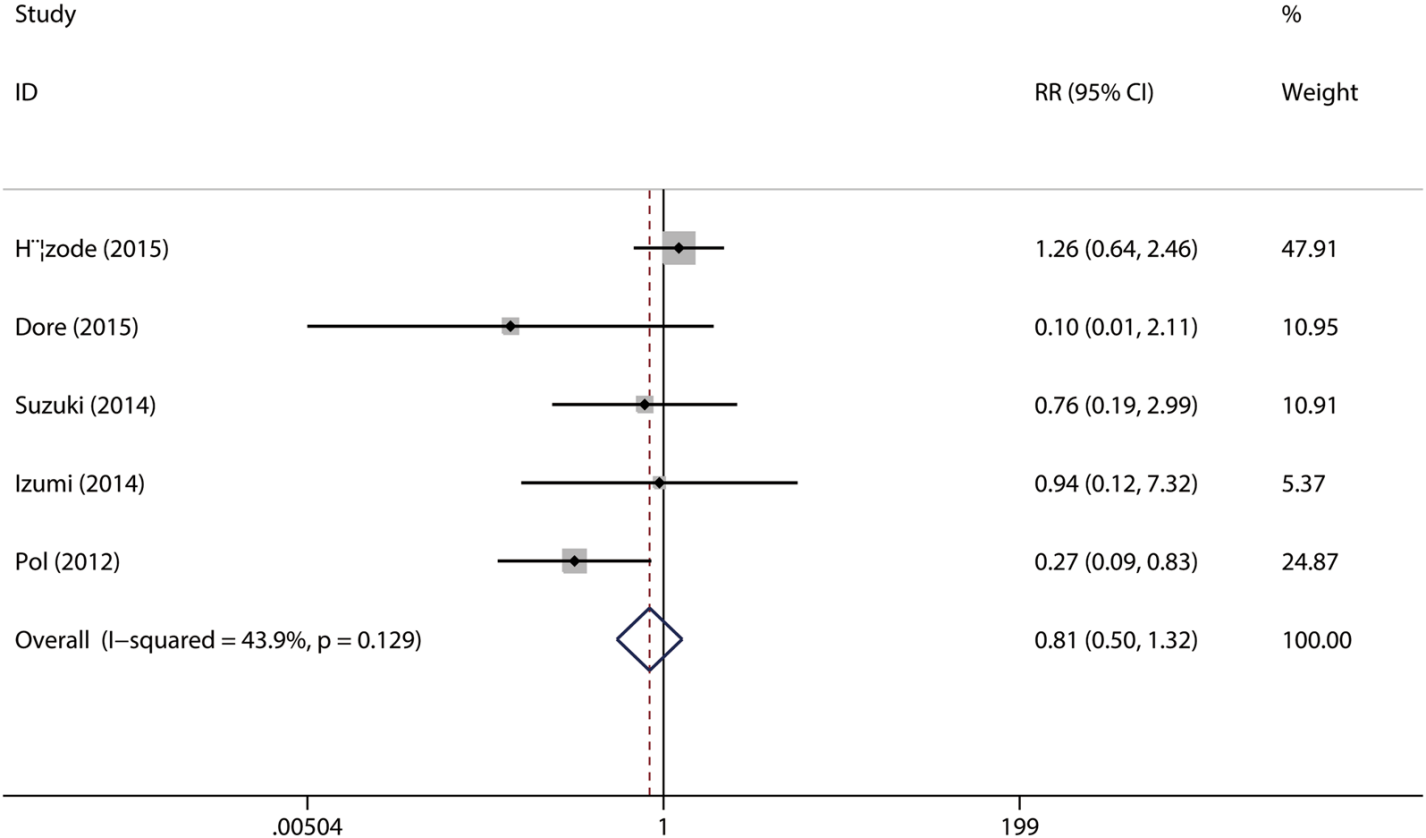
Forest plot of relapse rate of DCV + P/R and PBO + P/R for CHC

*Relapse* was defined as detectable HCV-RNA during 24 week follow-up after undetectable HCV-RNA at end of treatment. A total of the five studies (Hézode et al. 2015; Dore et al. 2015; Suzuki et al. 2014; Izumi et al. 2014; Pol et al. 2012) were included in this combined analysis. The test for the heterogeneity showed that there was no statistical significance (*I*^2^ = 43.9 %), so the fixed-effects model was used. Meta-analysis results revealed that there were no significant differences between the two groups in the relapse rate (*p* = 0.40 > 0.05, Fig. 4). Sensitive analysis showed that no individual studies could change the pooled results. Publication bias did not exist (*p* = 0.624) when the Begger test was performed.

#### Treatment discontinuation due to an adverse event (TDAE)

**Fig. 5:**
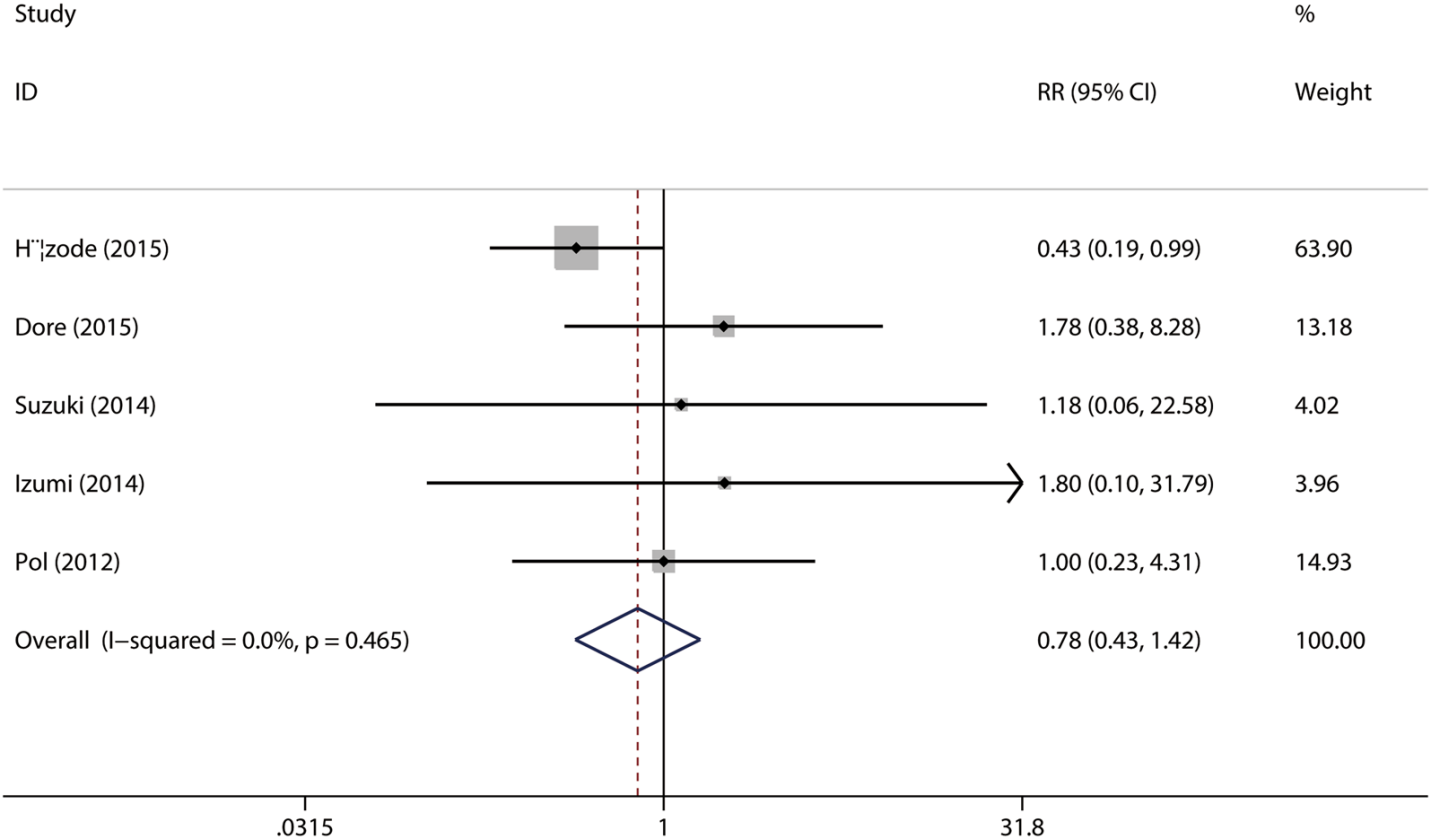
Forest plot of TDAE rate of DCV + P/R and PBO + P/R for CHC

TDAE was defined as subjects who stopped all study drugs due to an adverse event. A total of the five studies (Hézode et al. 2015; Dore et al. 2015; Suzuki et al. 2014; Izumi et al. 2014; Pol et al. 2012) were included in this combined analysis. The test for the heterogeneity showed that there was no statistical significance (*I*^2^ = 0 %), so the fixed-effects model was used. Meta-analysis results indicated that there were no significant differences between the two groups in the TDAE rate (*p* = 0.42 > 0.05, Fig. 5). Sensitive analysis showed that no individual studies could change the pooled results. Publication bias did not exist (*p* = 0.624) when the Begger test was performed.

### Subgroup analysis

#### Effect of high-dose (60 mg/day) use of daclatasvir on CHC

##### The RVR rate of daclatasvir (60 mg/day)

**Fig. 6:**
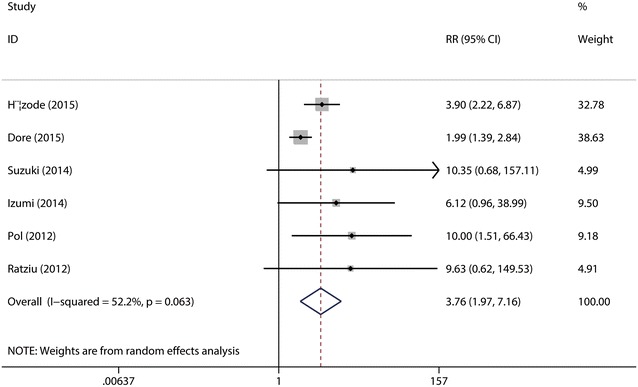
Forest plot of RVR rate of DCV (60 mg/day) + P/R and PBO + P/R for CHC

It was defined as the rate of patients who had a HCV viral load below the limit of quantitation or detection at week 4 of treatment with daclatasvir (60 mg/day). A total of six studies (Hézode et al. 2015; Dore et al. 2015; Suzuki et al. 2014; Izumi et al. 2014; Pol et al. 2012; Ratziu et al. 2012) were included in the subgroup. The test for the heterogeneity among the studies showed statistical significance (*I*^2^ = 52.2 %), so the random-effects model was used. Combined analysis suggested that the RVR rate was significantly higher in daclatasvir (60 mg/day) group (49.90 %) as compared with that in control group (13.97 %) (RR = 3.76, 95 % CI 1.97–7.16, *p* < 0.00001, Fig. 6). Sensitive analysis showed that no individual studies could change the pooled results. Publication bias did not exist (*p* = 0.851) when the Begger test was performed.

##### The SVR_24_ rate of daclatasvir (60 mg/day)

**Fig. 7:**
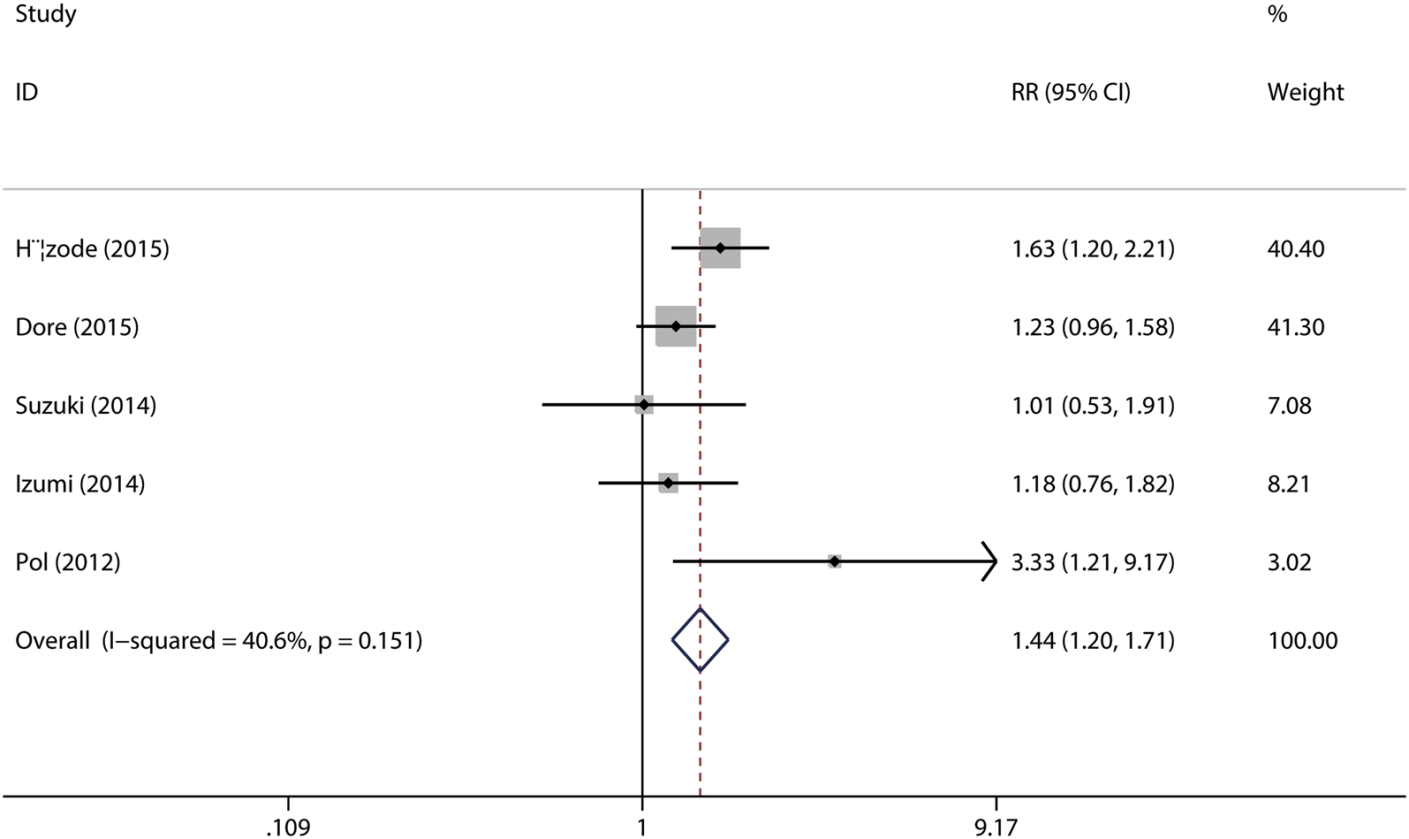
Forest plot of SVR rate of DCV (60 mg/day) + P/R and PBO + P/R for CHC

It was defined as the rate of patients who had a negative HCV RNA test 24 weeks after the end of treatment with daclatasvir (60 mg/day). A total of five studies (Hézode et al. 2015; Dore et al. 2015; Suzuki et al. 2014; Izumi et al. 2014; Pol et al. 2012) were included in the subgroup. The test for heterogeneity showed that there was no statistical significance (*I*^2^ = 40.6 %), so the fixed-effects model was used. Combined analysis revealed that the SVR_24_ rate was significantly higher in daclatasvir (60 mg/day) group (68.95 %) as compared with that in control group (47.77 %) (RR = 1.44, 95 % CI 1.21–1.71, *p* < 0.0001, Fig. 7). Sensitive analysis showed that no individual studies could change the pooled results. Publication bias did not exist (*p* = 0.327) when the Begger test was performed.

#### Effect of low-dose (10 mg/day) use of daclatasvir on CHC

##### The RVR rate of daclatasvir (10 mg/day)

**Fig. 8:**
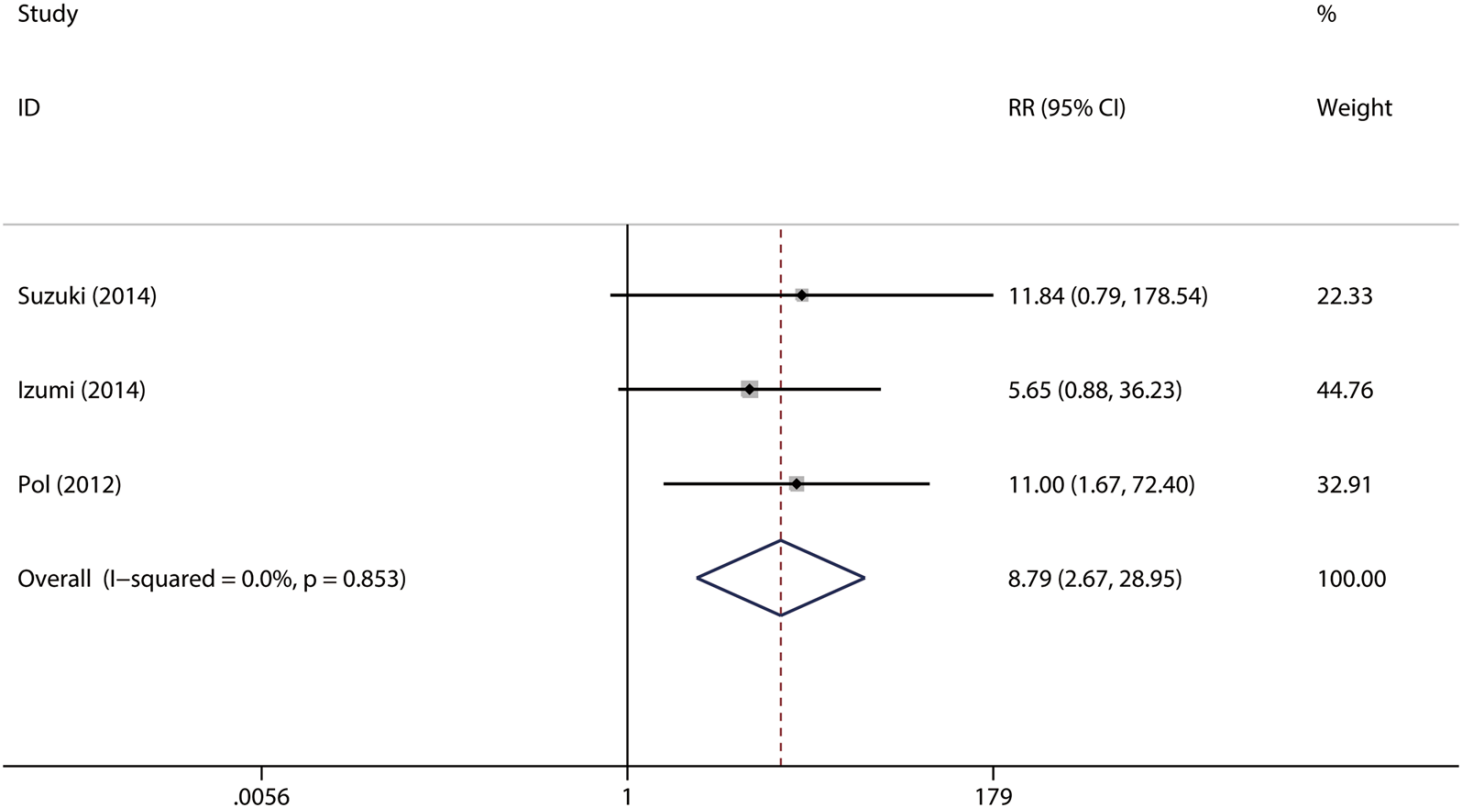
Forest plot of RVR rate of DCV (10 mg/day) + P/R and PBO + P/R for CHC

It was defined as the rate of patients who had a HCV viral load below the limit of quantitation or detection at week 4 of treatment with daclatasvir (10 mg/day). A total of three studies (Suzuki et al. 2014; Izumi et al. 2014; Pol et al. 2012) were included in the subgroup. The test for heterogeneity showed that there was no statistical significance (*I*^2^ = 0 %), so the fixed-effects model was used. Combined analysis suggested that the RVR rate was significantly higher in daclatasvir (10 mg/day) group (74.47 %) as compared with that in control group (7.14 %) (RR = 8.79, 95 % CI 2.67–28.95, *p* < 0.0001, Fig. 8). Sensitive analysis showed that no individual studies could change the pooled results. Publication bias did not exist (*p* = 0.602) when the Begger test was performed.

##### The SVR_24_ rate of daclatasvir (10 mg/day)

**Fig. 9:**
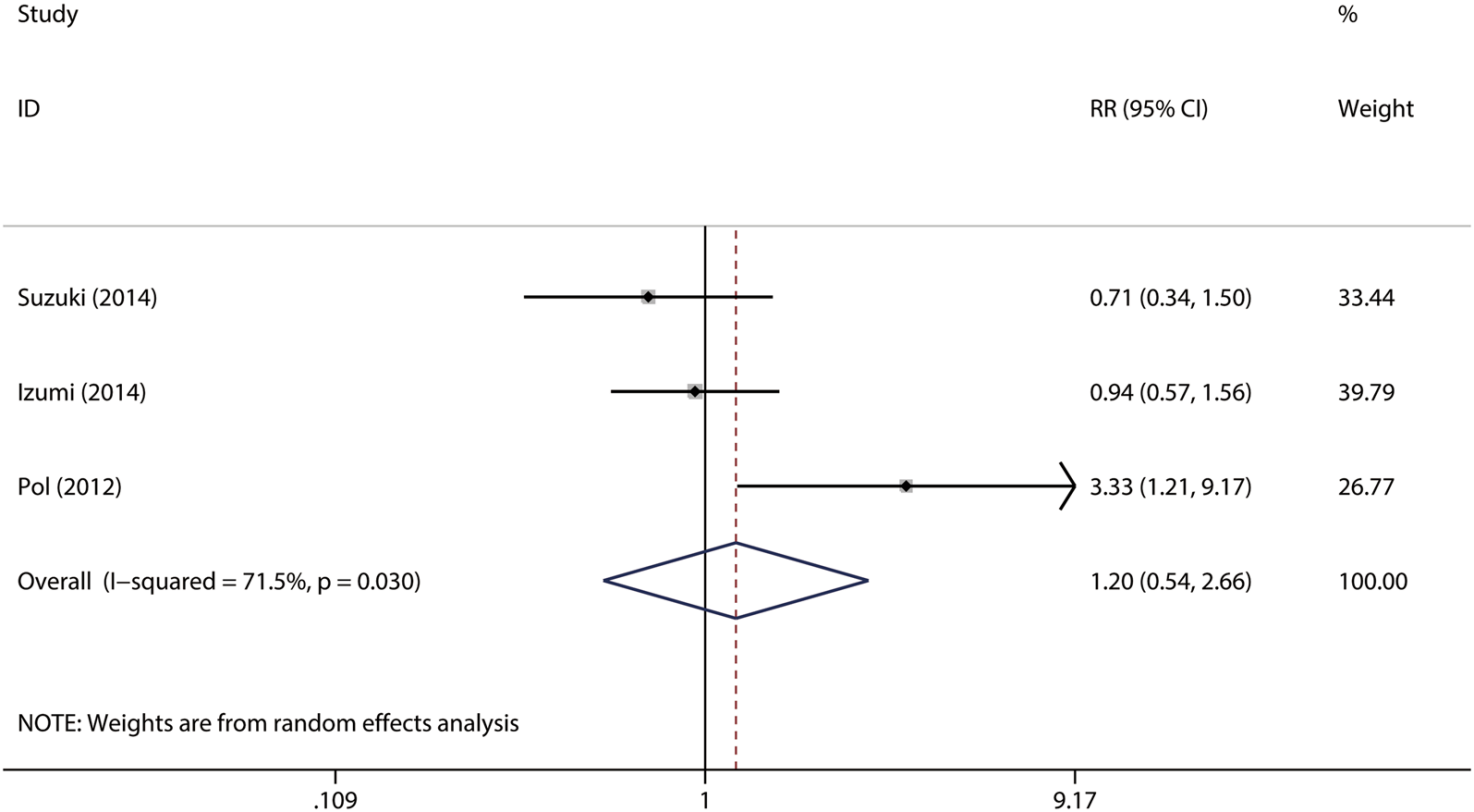
Forest plot of SVR rate of DCV (10 mg/day) + P/R and PBO + P/R for CHC

It was defined as the rate of patients who had a negative HCV RNA test 24 weeks after the end of treatment with daclatasvir (10 mg/day). A total of three studies (Suzuki et al. 2014; Izumi et al. 2014; Pol et al. 2012) were included in the subgroup. The test for the heterogeneity among the studies showed statistical significance (*I*^2^ = 71.5 %), so the random-effects model was used. Combined analysis revealed that there were no significant differences between the two groups in the SVR_24_ rate (63.83 vs. 50.00 %) (*p* = 0.65 > 0.05, Fig. 9). Sensitive analysis showed that no individual studies could change the pooled results. Publication bias did not exist (*p* = 0.602) when the Begger test was performed.

### Safety analysis

#### Nonspecific adverse events

Four clinical trials (Hézode et al. 2015; Suzuki et al. 2014; Izumi et al. 2014; Pol et al. 2012) mentioned nonspecific adverse events, which were composed of fatigue, headache, insomnia, nausea, diarrhea, decreased appetite, cough, arthralgia, etc. Meta-analysis results indicated that there were no significant differences in the nonspecific adverse events between daclatasvir and control groups (*p* > 0.05, Table 3). Sensitive analysis showed that no individual studies could change the pooled results. Publication bias did not exist (*p* > 0.05) when the Begger test was performed.

**Table 3:** Detailed adverse events of the included studies

Category	Concrete forms	Events/total (incidence rate, %)		RR (95 % CI)	*p* values
		DCV + P/R	PBO + P/R		
Nonspecific AEs	Fatigue	211 (49.76)	62 (58.49)	0.86 (0.72–1.03)	0.11
	Headache	184 (43.40)	48 (45.28)	0.96 (0.76–1.21)	0.73
	Insomnia	132 (31.13)	36 (33.96)	0.86 (0.64–1.14)	0.29
	Nausea	126 (32.31)	29 (29.59)	0.79 (0.37–1.68)	0.53
	Diarrhea	90 (23.20)	54 (57.45)	0.52 (0.20–1.36)	0.18
	Decreased appetite	104 (24.53)	26 (24.53)	0.99 (0.69–1.44)	0.98
	Cough	72 (18.60)	53 (54.08)	0.66 (0.18–2.37)	0.52
	Arthralgia	66 (17.01)	24 (25.53)	0.67 (0.44–1.01)	0.06
Liver dysfunction	Elevated ALT	9 (1.84)	1 (0.69)	1.51 (0.34–6.68)	0.59
	Elevated bilirubin	4 (0.82)	1 (0.69)	0.87 (0.19–4.07)	0.86
Hematologic abnormalities	Anemia	45 (8.59)	14 (8.92)	0.92 (0.53–1.59)	0.77
	Thrombocytopenia	12 (2.46)	6 (4.14)	0.63 (0.24–1.65)	0.34
	Neutropenia	139 (26.53)	46 (29.30)	0.89 (0.67–1.19)	0.44
Skin abnormalities	Rash	147 (28.05)	46 (29.30)	0.93 (0.71–1.23)	0.63
	Pruritus	179 (34.16)	49 (31.21)	1.06 (0.81–1.38)	0.67
	Alopecia	116 (27.36)	23 (21.70)	1.06 (0.50–2.24)	0.88

*AE* adverse event, *DCV* daclatasvir, *P* pegylated interferon-α, *R* ribavirin, *PBO* placebo

#### Liver dysfunction

Four clinical trials (Hézode et al. 2015; Dore et al. 2015; Suzuki et al. 2014; Izumi et al. 2014) mentioned the effect of daclatasvir on the liver function, which included elevated alanine aminotransferase (ALT), elevated total bilirubin (TBil), etc. Meta-analysis results indicated that there were no significant differences in the liver dysfunction between daclatasvir and control groups (*p* > 0.05, Table 3). Sensitive analysis showed that no individual studies could change the pooled results. Publication bias did not exist (*p* > 0.05) when the Begger test was performed.

#### Hematologic abnormalities

Five clinical trials (Hézode et al. 2015; Dore et al. 2015; Suzuki et al. 2014; Izumi et al. 2014; Pol et al. 2012) mentioned the effect of daclatasvir on the hematologic system, which included anemia, thrombocytopenia, neutropenia, etc. Meta-analysis results indicated that there were no significant differences in the hematologic abnormalities between daclatasvir and control groups (*p* > 0.05, Table 3). Sensitive analysis showed that no individual studies could change the pooled results. Publication bias did not exist (*p* > 0.05) when the Begger test was performed.

#### Skin abnormalities

Five clinical trials (Hézode et al. 2015; Dore et al. 2015; Suzuki et al. 2014; Izumi et al. 2014; Pol et al. 2012) mentioned the effect of daclatasvir on the skin, which included rush, pruritus, alopecia, etc. Meta-analysis results indicated that there were no significant differences in the skin abnormalities between daclatasvir and control groups (*p* > 0.05, Table 3). Sensitive analysis showed that no individual studies could change the pooled results. Publication bias did not exist (*p* > 0.05) when the Begger test was performed.

## Discussion

Hepatitis C virus (HCV) is divided to six genotypes and 70 subtypes, of which HCV genotype 1–3 infection occupy a leading position, and HCV genotype can influence disease characteristics, treatment options, and therapeutic response rates (Chinese Society of Hepatology et al. 2015; Ghany et al. 2009a, b). Due to no immunity and repeated infection, CHC is a serious viral infectious disease to human health and very difficult to prevent (World Health Organization 2016). Before the direct antiviral agents (DAA) emerged, the treatment of CHC, a regimen of PEG-IFN-α2a or 2b, and ribavirin (RBV) remains unsatisfactory, particularly in the large number of patients with HCV genotype 1 infection, whose sustained viral response rates are currently 40 % (Ghany et al. 2009a, b; European Association for the Study of the Liver 2014a, b).

Treatment for HCV infection is rapidly evolving with the introduction of direct-acting antiviral (DAA) agents (Li 2014; Yang and Wang 2014). The Guidelines Development Group recommends that persons with genotype 1 HCV infection should be considered for treatment with the currently approved direct-acting antivirals (telaprevir, boceprevir or simeprevir), given in combination with PEG-IFN and RBV rather than only PEG-IFN and RBV (European Association for the Study of the Liver 2014a, b). However, these drugs have to be taken three times daily and have a high pill burden, and are associated with adverse events, including skin rash and anaemia, which might reduce tolerability and adherence (World Health Organization 2016).

As far as we know, all-oral, interferon-free combinations of drugs are expected to cure more than 90 % of HCV infections and become a hot issue. Even so, a number of unsolved scientific questions remain. Firstly, IFN-based regimens are generally cheaper than combinations of DAAs without IFN. IFN-based regimens thus could be imposed as first line therapies in some settings (Chen 2016). Secondly, DAAs like sofosbuvir, ledipasvir and grazoprevir have not enter developing countries, such as China. Thirdly, the results of IFN-based therapies depend mainly on the patients’ responsiveness to IFN, which is determined genetically, the absence or presence of cirrhosis, and the HCV genotype. However, IFN-free regimens are not yet available or efficacious enough in some subsets of patients (Jean 2014). Fourthly, viral resistance will become an issue for patients who do not respond to all-oral, IFN-free regimens. When the drugs are approved, erroneous prescriptions, treatment of more difficult-to-cure, real-life patients, and/or suboptimal adherence to therapy will generate more frequent treatment failures, owing to selection of viruses that are resistant to the different classes of drugs (Jean 2014). Fifthly, if patients with decompensated cirrhosis or combined with other systemic disease need continuous treatment, DAA combinations will increase the risk of drug–drug interaction (Chen 2016). In summary, at present, patients in developing countries need some acceptable treatment options.

Daclatasvir (DCV), a new oral antiviral drug for CHC, is a HCV NS5A inhibitor. NS5A is a RNA-binding multifunctional viral protein and is essential for viral proliferation by interacting with other HCV nonstructural proteins and cellular proteins (Keating 2016; Scheel and Rice 2013; Qiu et al. 2011). Studies (Fridell et al. 2011; Lee et al. 2011; Wang et al. 2012) have demonstrated that DCV exerts antiviral activity by blocking NS3 protease-mediated cleavage of the viral polyprotein, altering the subcellular localisation of NS5A, preventing NS5A hyperphosphorylation, and inhibiting the formation of viral replication complexes. DCV supports once-daily dosing and covers HCV genotype 1–4 infections.

In the present meta-analysis, we included the six high quality published RCTs with adult CHC patients (Jadad scores ≥ 4) by searching several English and Chinese databases and reviewing relevant articles, showing some evidence for better therapeutic effect of daclatasvir plus the current standard therapeutic regimen (pegIFN-α/RBV) on CHC, especially HCV genotype 1 infection. Daclatasvir-based combination therapy yielded a significantly higher probability of achieving the overall RVR (46.43 vs. 18.97 %) with pooled RR of 3.77 (95 % CI 1.95–7.28, *p* < 0.0001) and a slightly higher probability of achieving the overall SVR_24_ (65.08 vs. 47.77 %) rate with pooled RR of 1.41 (95 % CI 1.18–1.68, *p* < 0.0001), and did not showed increased adverse events compared with the current standard therapeutic regimen. Further, in the subgroup analysis of high-dose daclatasvir (60 mg/day) group, the present meta-analysis showed the rate of RVR (49.90 %) with pooled RR of 3.76 (95 % CI 1.97–7.16, *p* < 0.0001) and the rate of SVR_24_ (68.95 %) with pooled RR of 1.44 (95 % CI 1.21–1.71, *p* < 0.0001) were slightly higher than those in the overall analysis. In the subgroup analysis of low-dose daclatasvir (10 mg/day) group, although daclatasvir-based combination regimen also indicated a significantly higher probability of achieving the RVR (74.47 vs. 7.14 %) with pooled RR of 8.79 (95 % CI 2.67–28.95, *p* < 0.0001), the SVR_24_ rate was similar between the daclatasvir and control groups (63.83 vs. 50.00 %) (*p* = 0.65). However, the subgroup analysis of low-dose daclatasvir (10 mg/day) group only included the three studies, and obviously the reliability of the results was poorer than high-dose daclatasvir (60 mg/day) group (including the six studies). The above results suggest that daclatasvir is a powerful and direct antiviral agents with improved RVR and SVR rates, oral route of delivery, once-daily administration, and less side effects. Therefore, it is recommended that daclatasvir, pegIFN and RBV triple therapy could be used as a treatment of CHC, especially HCV genotype 1 infection. As far as we know, this was the first meta-analysis that revealed that daclatasvir in combination with peg-IFN-α/RBV could achieve satisfactory therapeutic effects.

There were limitations to our meta-analysis that should be considered. The main limitation of this study was a small number of included RCTs and patients (six RCTs and 1100 patients). Secondly, the course of treatment were not consistent among the six RCTs with large span (12–48 weeks). Thirdly, daclatasvir emerged late, and the observation time of the included RCTs was 24 weeks with a lack of longer follow-up information, so the long-term efficacy was unclear. Fourthly, HCV genotype 1 infection accounted for 73.1 %, genotype 2–3 infection 22.2 %, and genotype 4 infection 4.7 % in the included 1100 adult patients. Due to the limited number of cases, we couldn’t provide subgroup analysis data on the basis of the four genotypes. Lastly, the limitation of possible publication bias should be taken into consideration because the study with positive results were easier to be reported, although possibility of publication bias did not existed using the Begger test.

In summary, this meta-analysis indicated that high-dose daclatasvir (60 mg/day) in combination with peg-IFN-α/RBV is effective and safe in treating adult patients with CHC, especially HCV genotype 1 infection. We believe daclatasvir in combination with peg-IFN-α/RBV could be respected as a natural bridge between the past (peg-IFN and ribavirin) and the current (IFN free direct antiviral agents), which has guiding significance to clinical work. To obtain an exact finding with respect to daclatasvir use of CHC treatment, additional high-quality, large sample and multicenter, randomized, double-blind, placebo-controlled clinical trials on this issue are needed.

## Abbreviations

CHC: chronic hepatitis C
HCV: chronic hepatitis C virus
RCT: randomized controlled trial
DAA: direct antiviral agent
NS3/4A: nonstructural protein 3/4A
DCV: daclatasvir
pegIFN-α: peginterferon-α
RBV: ribavirin
placebo: PBO
RVR: rapid virological response
SVR_24_: sustained virological response at post-treatment week 24
VB: virological breakthrough
TDAE: treatment discontinuation due to an adverse event
SAE: serious adverse event
ALT: alanine aminotransferase
TBil: total bilirubin
RR: risk ratio
CI: confidence interval
